# Supplementary material for: CDK1 and PLK1 coordinate the disassembly and reassembly of the nuclear envelope in vertebrate mitosis
Source: Oncotarget. 2017 Dec 23;9(8):7763–73. doi: 10.18632/oncotarget.23666 (PMC5814256; doi:10.18632/oncotarget.23666)
Supplement: Supplementary file 1 [file oncotarget-09-7763-s001.pdf]

CDK1 and PLK1 co-ordinate the disassembly and re-assembly of the nuclear envelope in vertebrate mitosis

SUPPLEMENTARY MATERIALS

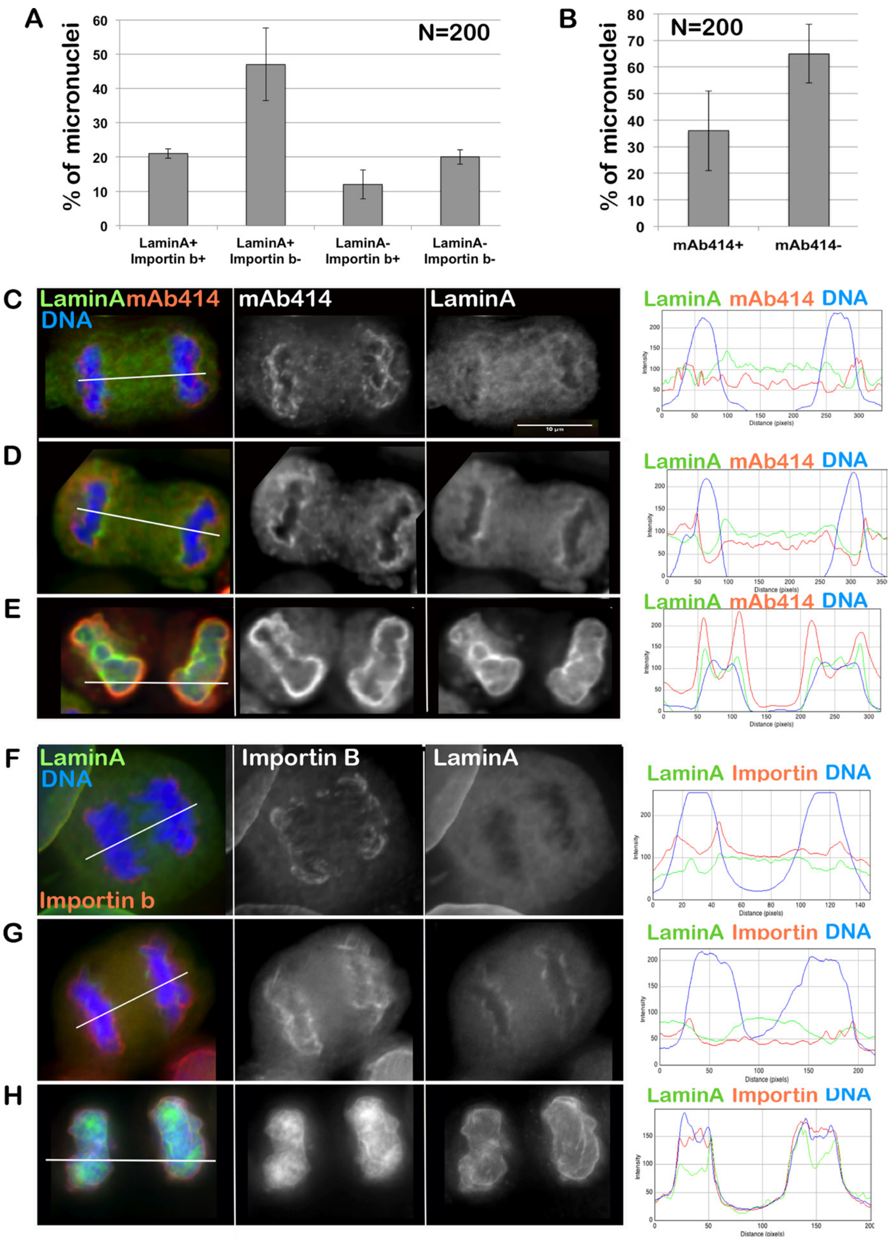

Supplementary Figure 1: Importin β and Nups are loaded before Lamin A/C on the anaphase chromosomes starting from the pole-ward side of the chromatin. (**A–B**) HeLa cells were stained for Importin β, mAb414 and Lamin A/C. The presence/absence of the staining in MN was recorded and plotted. The data are presented as mean of 3 independent experiments. The error bars indicate the SD between 3 replicates. (**C–H**) HeLa cells were stained for mAb414 (C–E) or Importin β (F–H) and Lamin A/C. Intensity profiles on the distribution of the markers along the anaphase cell are shown. In early anaphase, mAb414 (C) and Importin β (F) accumulate around the chromosomes starting from the side toward the pole but no signal is present on the telomeric side (the middle of the cell) of the chromosomes; at this stage the Lamina is still diffused; later in anaphase mAb414 (D) and Importin β (G) completely surround the chromatin and the lamina starts loading. In cytokinesis both NM components are fully assembled onto the chromosomes (E and H).

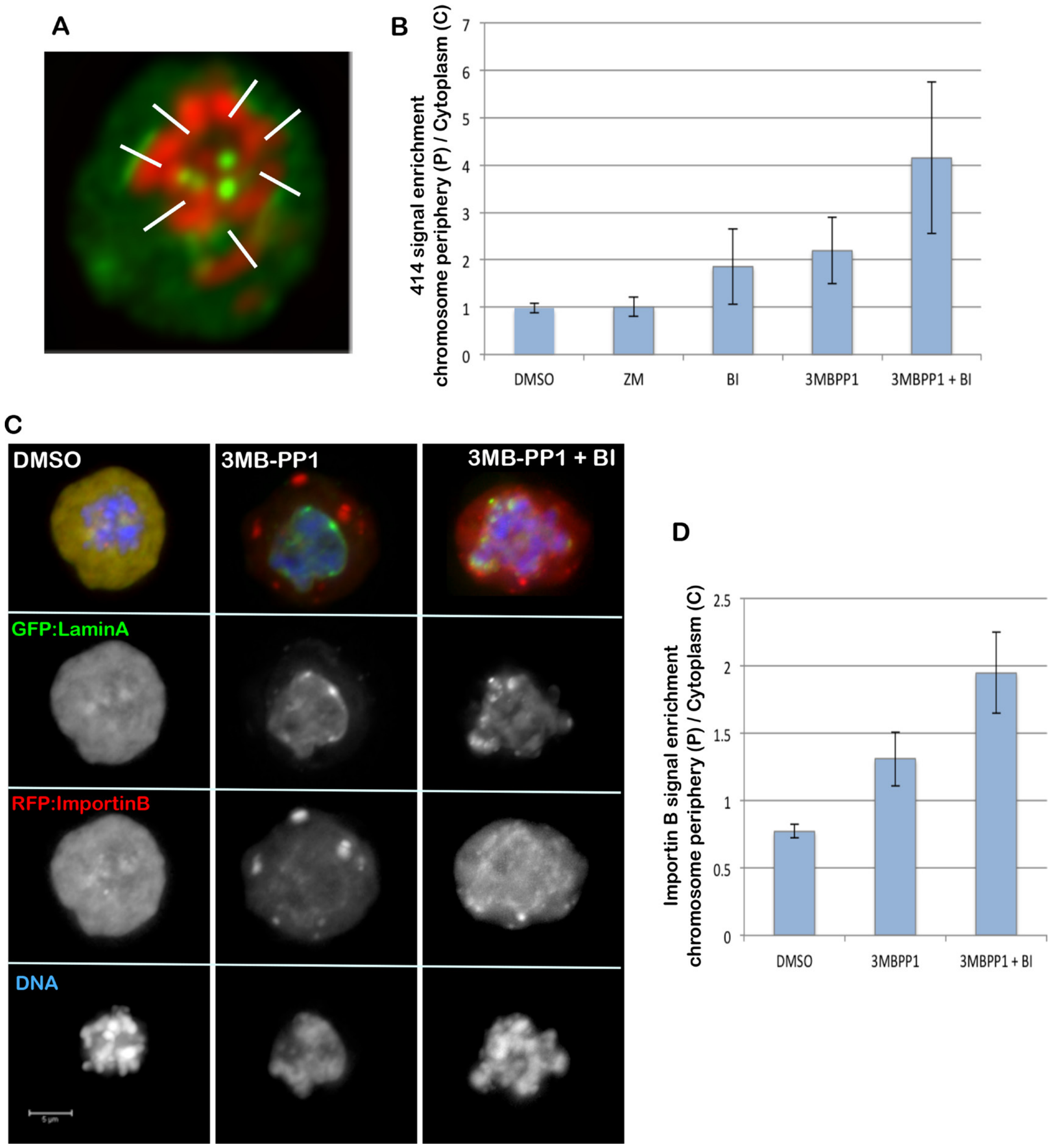

Supplementary Figure 2: Localisation of NE components during kinase inhibition. (**A**) Example of enrichment measurements on chromosome periphery versus cytoplasm. Line scans were randomly generated around the chromosomes and the ration between the intensity at the chromosome periphery (P) and the cytoplasm (C) was calculated. (**B**) Average enrichment of mAb414 on the peripheral chromatin versus cytoplasm (measured as shown in A) in DT40CDK1^{AS} arrested with nocodazole and treated with DMSO, BI2536 (BI), ZM447439 (ZM), 3MB-PP1 or 3MB-PP1+BI2536 (BI+3MB-PP1) (Figure 3C); The data are presented as mean and the error bars indicate the SD. (**C**) DT40CDK1^{AS} were transfected with GFP:Lamin or RFP:Importin β and arrested in nocodazole for 4h then treated for 15 min with DMSO, 3MB-PP1 or 3MB-PP1+BI2536. Scale bar 5 µm. (**D**) Enrichment of Importin β at the peripheral chromatin versus cytoplasm as in (B).

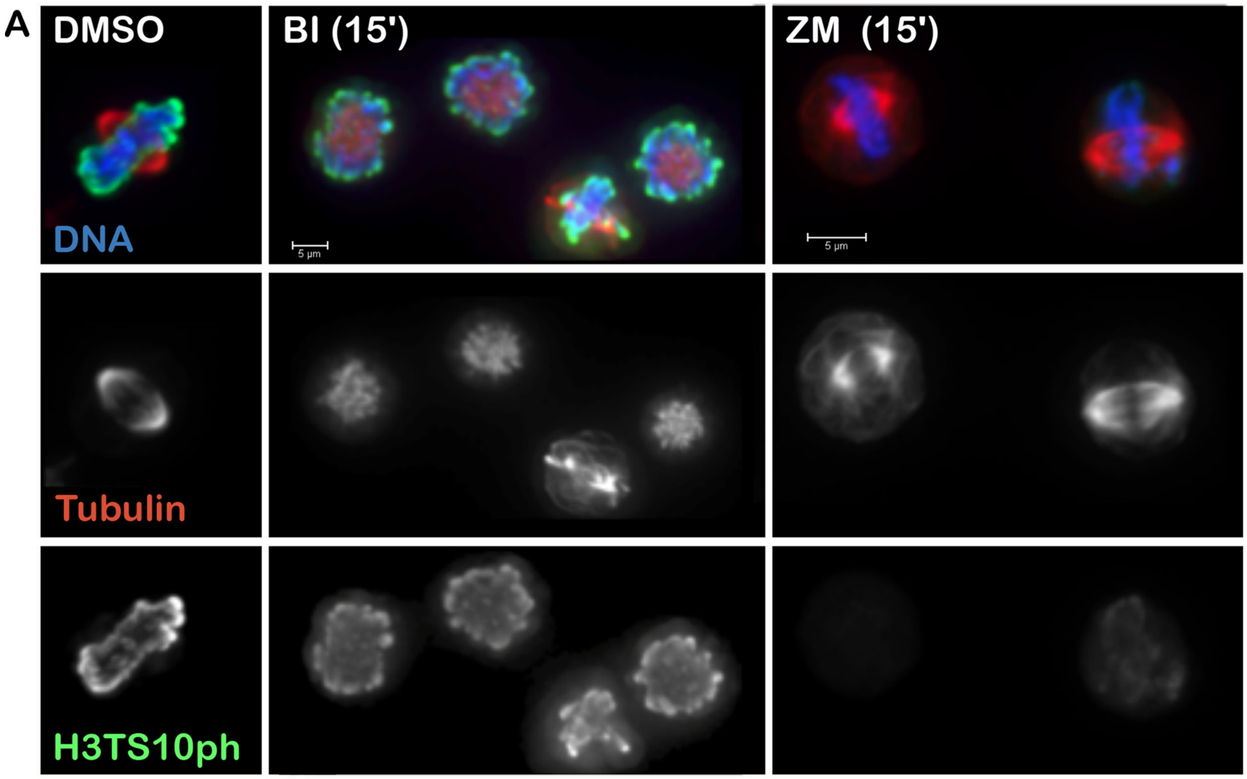

Supplementary Figure 3: Short inhibition of PLK and Aurora B. DT40CDK1AS cells were blocked with 3MB-PP1 for 6 h then released for 60' in MG132 containing medium. After BI or ZM were added for 15'. The cells were then fixed and stained with anti atubulin and anti H3S10ph antibodies.
